# Cultural beliefs, utility values, and health technology assessment

**DOI:** 10.1186/s12962-018-0103-1

**Published:** 2018-06-01

**Authors:** Jörg Mahlich, Piyameth Dilokthornsakul, Rosarin Sruamsiri, Nathorn Chaiyakunapruk

**Affiliations:** 1 Health Economics, Janssen Pharmaceutical KK, 5-2, Nishi-kanda 3-chome Chiyoda-ku, Tokyo, 101-0065 Japan; 2 Düsseldorf Institute for Competition Economics (DICE), University of Düsseldorf, Düsseldorf, Germany; 3 Center of Pharmaceutical Outcomes Research, Naresuan University, Phitsanulok, Thailand; 4 School of Pharmacy, Monash University Malaysia, Subang Jaya, Malaysia

## Abstract

**Background:**

Health-care utilities differ considerably from country to country. Our objective was to examine the association of cultural values based on Hofstede’s cultural dimensions’ theory with utility values that were identified using the time trade off method.

**Methods:**

We performed a literature search to determine preference-based value algorithms in the general population of a given country. We then fitted a second-order quadratic function to assess the utility function curve that links health status with health-care utilities. We ranked the countries according to the concavity and convexity properties of their utility functions and compared this ranking with that of the Hofstede index to check if there were any similarities.

**Results:**

We identified 10 countries with an EQ-5D-5L-based value set and 7 countries with an EQ-5D-3L-based value set. Japan’s degree of concavity was highest, while Germany’s was lowest, based on the EQ-5D-3L and EQ-5D-5L value sets. Japan also ranked first in the Hofstede long-term orientation index, and rankings related to the degree of concavity, indicating a low time preference rate.

**Conclusions:**

This is the first evaluation to identify and report an association between different cultural beliefs and utility values. These findings underline the necessity to take local values into consideration when designing health technology assessment systems.

## Background

Utility is a preference weight, where preference can be measured in terms of value or desirability [1]. Accordingly, health-care utilities need to be built on preferences for the different health statuses. The more desirable health statuses generally receive a greater utility value. Utility is measured on an interval scale of 0–1, where 0 indicates death and 1 indicates full health status with negative values assigned to states worse than death [2].

Utility value sets are country-specific and there are huge differences between countries. The reasons for these differences are mostly unclear. A recent study [3] found that the utility value as measured by EuroQOL-5D-3L in the Thai population at age 60 was nearly 0.65, while that of the Japanese population was found to be 0.91 (40% higher). One possible explanation is that the quality of health status in Thai people is simply not as high as that of the Japanese. But even based on comparable health statuses, higher utility values are reported for the Japanese population. Our hypothesis is that this is probably due to the way utilities are identified, which ultimately reflects cultural values and beliefs. Take the EQ-5D, for example, where utilities for a small number of health statuses are obtained by means of a time trade-off (TTO) exercise and later on generalized with regression techniques to the remaining health states. Using the TTO concept, respondents are asked to choose either to live 10 years in a specified current health status or to give up some life years to live for a shorter period in full health. The number of years in full health that are deemed of equal value to 10 years in the current health state describes the utility value [4]. If the respondent is not willing to give up life years against full health at all, then the utility value of the current health state is 1.

It is evident that the willingness to trade lengths of life for quality of life reflects a person’s time preference which in turn is a cultural value. The Dutch sociologist Geert Hofstede classified cultures according to their short-term or long-term planning horizon. In his “Confucian dimension”, the Asian cultures of China, Hong Kong, Taiwan and Japan have the longest-term orientations while many Western countries such as the US and UK only achieve ranks 17 and 18, respectively [5, 6]. If time preference is determined by cultural values, then utilities obtained by TTO have a cultural dimension as well. A person with a low preference for the present, which is equivalent to a long-term orientation, would be less willing to trade lengths of life for quality of life and would therefore have a higher utility value than a person from a culture with a strong preference for the present seeking gratification in the here and now. Of note, such differences would occur in the same health states due to differences in cultural values with far reaching implications for health technology assessment. If the higher utility values of Asians simply result from their long-term orientation, a medical intervention likely to restore perfect health in an Asian context will not create as much value (regarding additional utilities) as the same intervention in a culture with a short-term orientation. This is because a culture with short-term orientation would value given health states lower and the potential utility increase of an intervention is larger. On the other hand, societies with long-term orientation would probably be more willing to pay for an additional year of life. However, this would be reflected in a higher Incremental cost-effectiveness ratio (ICER) threshold and should not be confused with the additional utilities gained from an intervention.

Similar arguments can be put forward for utilities derived from standard gamble (SG) known as the gold standard [7] in utility identification since it is derived from Neumann–Morgenstern’s rigorous axiom-based economic decision theory under uncertainty [8]. Here, a utility value equals the probability value that makes a person indifferent to a certain outcome (health state) and a gamble in which-probability (1 − p)—means the person dies immediately and—probability p-means perfect health is restored. Culture here also plays a critical role in terms of risk aversion. Again, one of Hofstede’s cultural dimensions—apart from long-term orientation—is the avoidance of uncertainty. This dimension focuses on how cultures adapt to changes and manage uncertainty. Emphasis is on the extent to which a culture feels threatened or is concerned about ambiguity. The US and UK are considered as countries with a culture of high risk-taking, while Japan or Italy are characterized by a high uncertainty avoidance score [9]. Followers of a risk averse society would hesitate to engage in the gambling process, which means that they would assign high utility to any given health state. Recently, it was shown that female students reported higher quality of life values when they were elicited by SG [10]. This outcome was found to be associated with lower risk preference by females, a well-established finding in behavioral economics [11–19]. One plausible explanation for this was proposed as the display of overconfidence exhibited by men about their decisions, especially when forecasting potential outcomes more precisely than women [20]. Not only do women and men have different attitudes towards risk, but societies as a whole. In terms of Hofstede’s risk avoidance ranking, countries such as Greece, Belgium, Italy, and Korea rank high while the US und the United Kingdom have very low scores on the uncertainty avoidance index.

To the best of our knowledge, there is no published study on the association of culture and health utility to date. Therefore, we aim to determine this association by assessing utility functions of different countries in terms of their curvature properties and profiles.

## Methods

To test our proposition that culture shapes utility values, our objective was to investigate whether utility functions that are based on the TTO method show different profiles that are in line with the time preference of each country. We also wanted to test our hypothesis that culturally determined risk attitudes shape utility functions when utilities are elicited by the SG method.

**Fig. 1:**
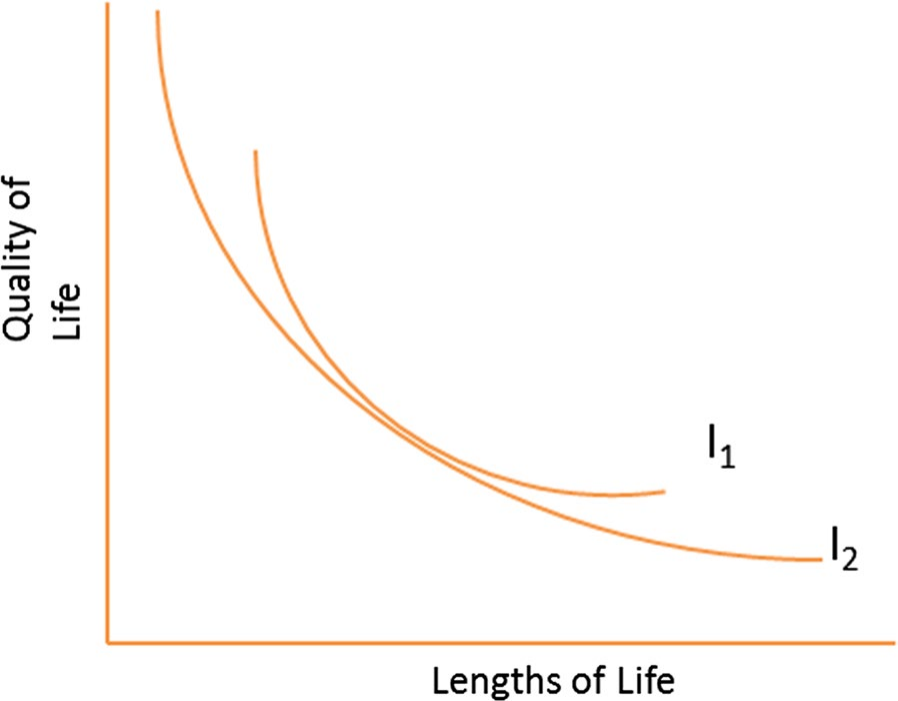
Indifference curves for low (I_1_) and high (I_2_) time preference

Regarding TTO, a country with a low time preference would have a very high marginal rate of substitution of life span and quality of life. The two indifference curves that depict a combination of two “goods”, namely lengths of life and quality of life that provide the same level of satisfaction to the consumer are presented in Fig. 1. I_1_ is an indifference curve for a country with a low time preference aka long-term orientation.

**Fig. 2:**
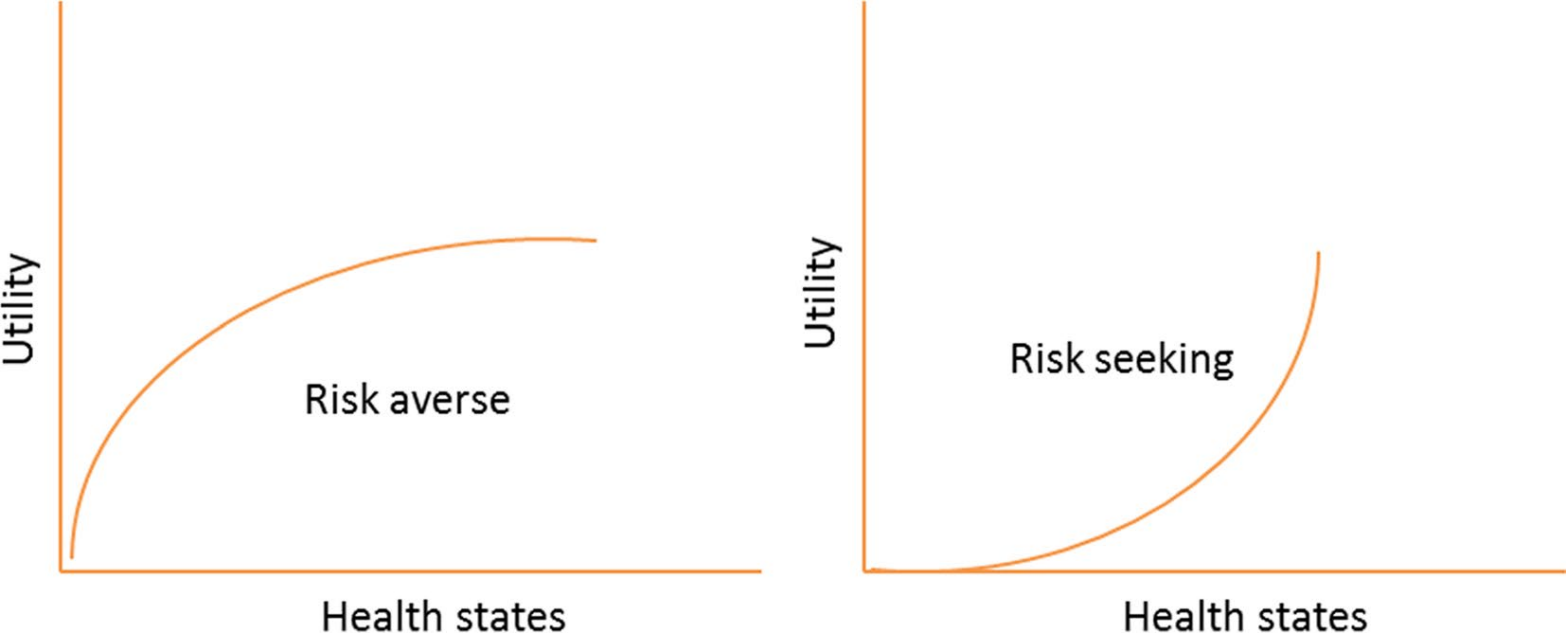
Utility functions according to risk preference

For people to trade off lengths of life against quality of life, one would need to offer higher values of quality of life for forgoing one unit of life span than in countries with a higher time preference (such as I_2_). The utility function that links different health states to utility values have different profiles as well. We would expect that utility functions from long-term orientation countries are more convex than those of short-term orientation countries. To illustrate this, imagine an individual whose long-term preference is so high that he would by no means trade a unit of his life span against quality. Consequently, his utility values will be one for all health states except death. The same argument can be developed for SG derived utilities. An extremely risk averse individual would never engage in the gamble and would attach the value of 1 for all health states except death, which is an extreme example of a convex utility function. A graphical representation of the utility functions according to risk preference is presented in Fig. 2.

We performed a literature search using PubMed to determine preference-based value algorithms derived from either standard gamble or time trade-off in the general population. The search terms were “utility” OR “preference” OR “standard gamble” OR “time trade off” OR “EQ-5D” OR “health related quality of life” OR “health utility index” OR “SF-36” OR “SF-6D” AND “healthy volunteer” OR “healthy population” OR “healthy”. The Euroqol and 36-item short form websites were searched as well in January 2017 [21, 22]. Most of the value sets for EQ-5D-3L were from Szende et al. [23]. The EQ-5D-3L value set from Thailand is from Tongsiri and Cairns [24], and the value set for the EQ-5D-5L can be found in Van Hout et al. [25]. Some of the value sets included in here were cross-walk value sets derived by means of a mapping approach to the three-level version of the EQ-5D.

The algorithms can be used to generate utility values of various sets of responses. We used country specific utility sets to fit a second-order quadratic function so that the utility function connecting health states and utilities can be estimated. To assess the curvature of this utility function, we then calculated its second derivative to determine whether the function is concave (i.e. negative sign of the second derivative) or convex (i.e. positive sign of the second derivative). This can be interpreted as a quasi-Arrow–Pratt measure, which uses the curvature of the utility functions to determine risk aversion [26]. Following that, we ranked the countries according to the concavity and convexity properties of their utility functions and compared their ranking with that of the Hofstede index to check if there were any similarities as hypothesized.

In the final step, we ran a scenario analysis to assess the practical implications of our findings. We constructed two scenarios for four countries that were characterized by a different culture based on Hofstede’s taxonomy, namely Japan (JP), Germany (GE), United Kingdom (UK), and the US.

The first scenario was a breakthrough innovation. In this scenario, we assumed that the hypothetical product could increase any EQ-5D-5L-health status from 5 (worst) to 1 (best). For example: before receiving treatment, the health status of a patient was 55,523 which corresponds to a utility value of 0.068 in JP, 0.085 in GE, − 0.142 in the UK, and 0.127 in the US [21]. We assumed that breakthrough treatment changes the health status to 11,123, thus changing the utility values to 0.721 in JP, 0.909 in GE, 0.75 in the UK, and 0.809 in the US. We then calculated the incremental utility for each change (JP 0.653, GE 0.824, UK 0.893 and US 0.682). We performed the same calculations for all possible health states and averaged the incremental change for this hypothetical product. Then, we calculated the ICER value based on the following assumptions. First, we assumed a drug price of 1,000,000 Japanese Yen (JPY), without any other incremental costs or cost offsets. Second, we assumed a time horizon of 1 year.

The second scenario we implemented was incremental innovation. Here, we assumed that the hypothetical product can improve the EQ-5D-5L-health status by only one unit in all five dimensions (e.g. from 55,523 to 44,412). The calculations were the same as in the breakthrough scenario.

## Results

We found four utility sets for countries where utilities were elicited by means of SG [27–29]. Since this number was too low for a meaningful analysis, only results for studies based on TTO were reported. We identified TTO based value sets for the following countries: The EQ-5D-5L based value sets consisted of 10 countries, namely, Japan, Denmark, France, Germany, the Netherlands, Spain, Thailand, UK, US, and Zimbabwe. Seven countries were identified from EQ-5D-3L, including Japan, Denmark, Germany, the Netherlands, Spain, Thailand, UK, and Zimbabwe.

The estimates of the 2nd order quadratic utility function are reported in Table 1. Depending on the second derivative of this function, the estimates were categorized as concave or convex.

**Table 1:** Second order quadratic utility functions

Country	2-nd order quadratic line	Tool	Interpretation	Rank (equation 5D-5D-5L)	Rank (EQ-5D-3L)
Japan	Y = − 0.0000000550x^2^ + 0.0003563210x − 0.0742209093	EQ-5D-5L	Concave	1	–
Japan	y = − 0.0000062x^2^ + 0.0045459x − 0.0090418	EQ-5D-3L	Concave	–	1
Denmark	Y = − 0.0000000446x^2^ + 0.0004197915x − 0.1655666889	EQ-5D-5L	Concave	2	–
Denmark	y = − 0.0000054x^2^ + 0.0057193x − 0.2949838	EQ-5D-3L	Concave	–	2
France	Y = 0.0000000237x^2^ + 0.0001943942x − 0.2303118192	EQ-5D-5L	Convex	6	–
Germany	Y = 0.0000000323x^2^ + 0.0001660183x + 0.0082592409	EQ-5D-5L	Convex	10	–
Germany	y = 0.0000094x^2^ + 0.0014504x – 0.0378014	EQ-5D-3L	Convex	–	8
Netherlands	Y = 0.0000000268x^2^ + 0.0001734792x − 0.0647394984	EQ-5D-5L	Convex	8	–
Netherlands	y = 0.0000055x^2^ + 0.0021384x − 0.0380753	EQ-5D-3L	Convex	–	5
Spain	Y = 0.0000000251x^2^ + 0.0002372594x − 0.2745223392	EQ-5D-5L	Convex	7	–
Spain	y = 0.0000083x^2^ + 0.0025337x − 0.3504985	EQ-5D-3L	Convex	–	7
Thailand	Y = − 0.0000000134x^2^ + 0.0002816214x − 0.1901163980	EQ-5D-5L	Concave	4	–
Thailand	y = 0.0000015x^2^ + 0.0033337x − 0.2652283	EQ-5D-3L	Convex	–	4
UK	Y = 0.0000000299x^2^ + 0.0002053902x − 0.2343214473	EQ-5D-5L	Convex	9	–
UK	y = 0.0000079x^2^ + 0.0023519x − 0.3067348	EQ-5D-3L	Convex	–	6
USA	Y = 0.0000000092x^2^ + 0.0001832790x + 0.0991735591	EQ-5D-5L	Convex	5	–
Zimbabwe	Y = − 0.0000000189x^2^ + 0.0002390259x + 0.1619338051	EQ-5D-5L	Concave	3	–
Zimbabwe	y = − 0.0000025x^2^ + 0.0034765x + 0.0751729	EQ-5D-3L	Concave	–	3

The ranking of concavity was based on the second derivative of each country. Lowest second derivative was ranked as the number 1 of the ranking

**Fig. 3:**
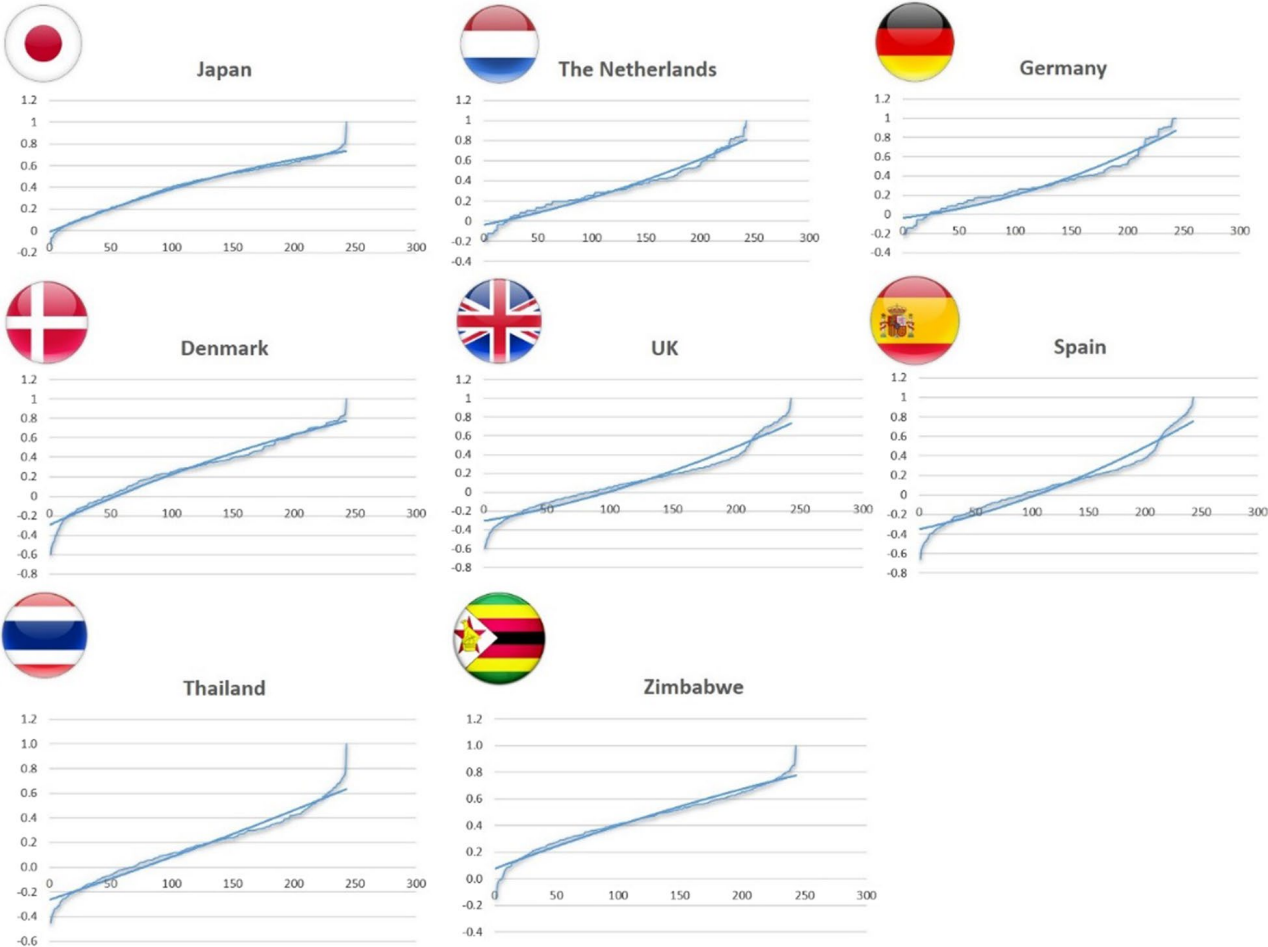
2nd-order quadratic predictions for longevity derived by EQ-5D-3L

**Fig. 4:**
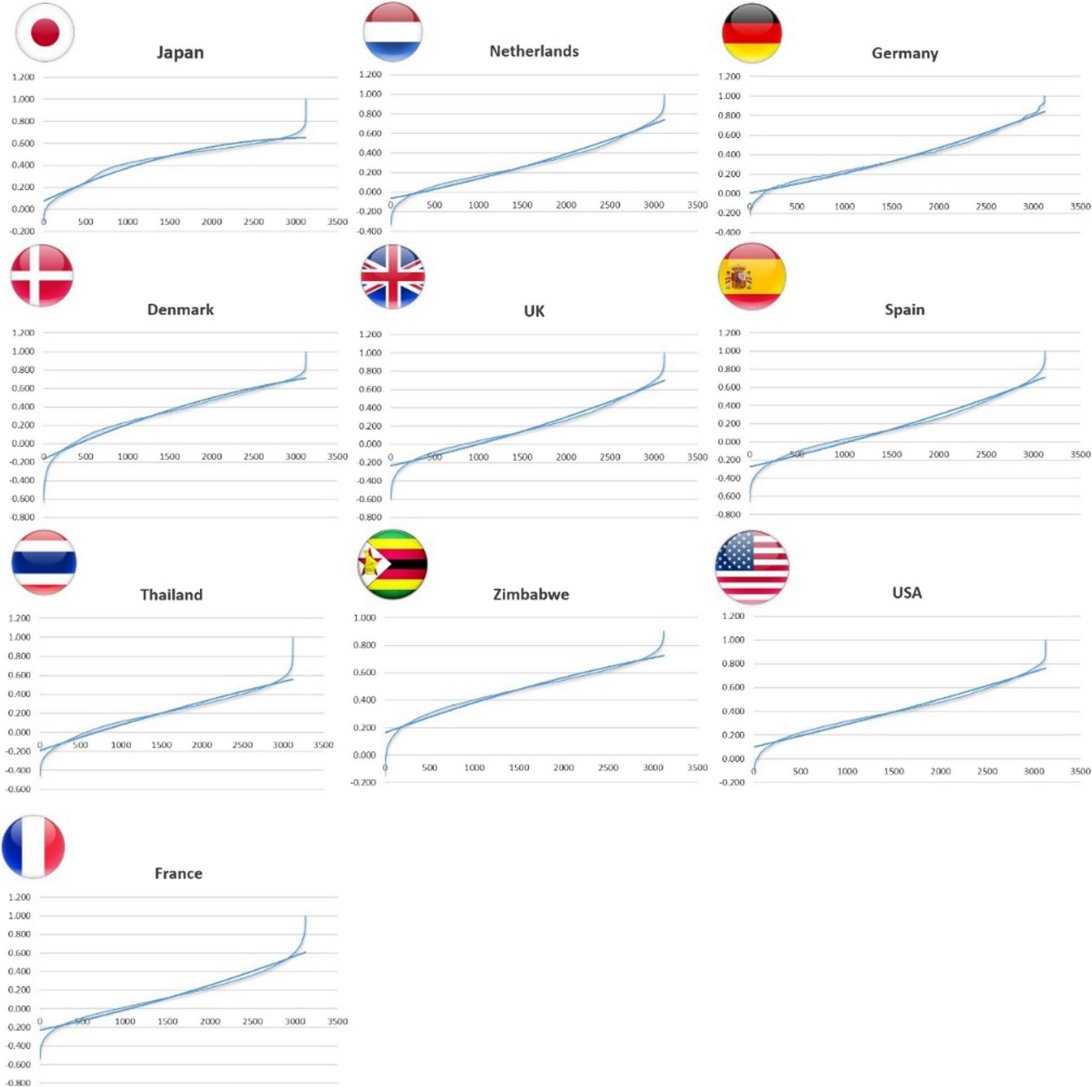
2nd-order quadratic predictions for longevity derived by EQ-5D-5L

The lowest second derivative was ranked as number 1 on a scale in descending order. As previously mentioned, the maximum rank indicates the highest degree of concavity. The more concave a function, the higher the long-term preference for the respective country. In addition, Figs. 3, 4 are graphical representations of the utility functions of the countries selected based on EQ-5D-3L and EQ-5D-5L value sets.

When using the EQ-5D-3L value set, it is evident that the concave relation between health status and utility was notable for Japan, Denmark, and Zimbabwe (Table 1 and Fig. 3), with Japan ranked as the country with the highest order of concavity. On the other hand, a convex relation was found for the Netherlands, Germany, UK, Spain, and Thailand, with Germany ranking as the country with the lowest order of concavity.

Based on EQ-5D-5L value set, the concave relation of health status and utility were found for Japan, Denmark, Zimbabwe, and Thailand (Fig. 4). Similar to the results from the EQ-5D-3L value set, Japan was ranked as the country with the highest order of concavity, indicating the highest degree of long-term preference, or, in other words, the lowest time preference rate. On the other hand, a convex relationship was detected for France, Germany, Netherlands, Spain, UK, and US. Once again, Germany was ranked as the country with the lowest degree of concavity.

The ranks in the Hofstede index [5, 30] as well as per utility function curvature are reported in Table 2.

**Table 2:** Long term orientation index of included countries. Source: Hofstede et al. (2010) [5]

Country	Long term orientation rank	EQ-5D-3L concavity rank	EQ-5D-3L 5L concavity rank
Japan	1	1	1
Germany	2	8	10
Netherlands	3	5	8
France	4	–	6
UK	5	6	9
Spain	6	7	7
Denmark	7	2	2
Thailand	8	4	4
USA	9	–	5
Zimbabwe	Not applicable	3	3

The results for Japan are fairly consistent in that they rank first in the Hofstede long-term orientation index as well as in both rankings representing the degree of concavity. Conversely, we found conflicting results for both Germany and Denmark. Although Germany has a high degree of long-term orientation, similar to Japan, this is not reflected in the shape of the EQ-5D utility functions. For Denmark, the opposite holds true: long term orientation (LTO) according to Hofstede is lower than the utility functions would suggest.

The results of the scenario analysis are reported in Table 3. We found that the ICER of both the breakthrough innovation as well as the incremental innovation is highest in Japan, indicating that the value it brings in terms of additional utilities is the lowest. Compared with the UK that has the lowest ICER in both scenarios, the value for Japan is twice as high.

**Table 3:** Results of scenario analysis

	Germany	Japan	UK	US
Break through innovation				
Incremental cost (JPY)	1,000,000	1,000,000	1,000,000	1,000,000
Incremental QALY	0.506	0.289	0.575	0.400
ICER (JPY per QALY)	1,976,121	3,463,208	1,738,434	2,499,378
Incremental innovation				
Incremental cost (JPY)	1,000,000	1,000,000	1,000,000	1,000,000
Incremental QALY	0.340	0.194	0.387	0.269
ICER (JPY per QALY)	2,939,257	5,151,130	2,585,724	3,717,543

*UK* United Kingdom, *US* United States; *QALY* quality-adjusted life year, *ICER* incremental cost-effectiveness ratio, *JPY* Japanese Yen

## Discussion

Our findings indicate that different utility values are associated with various cultural beliefs which may impact cost-utility analysis. We also found a low level of evidence that countries with a long-term horizon (according to Hofstede) show a more concave profile of utility function. Paradoxically, a social planner with a restricted budget who aims at maximizing the global welfare (sum of utilities) would potentially distribute less budget to countries with a low time preference rate. The reason is that those countries already have higher utility values, resulting in a smaller scope for improvement, because, by definition, utility values are capped at 1. Only in health states that close to death are the slopes of the utility functions of low time preference rate countries like Japan higher, as are marginal utilities when health is improving. Such countries would therefore benefit more from interventions providing only marginal improvement of a patient’s health state instead of restoring perfect health. Other countries with a high time preference would considerably benefit from interventions providing restoration of a perfect health state (utility level 1). The results of the scenario analyses further support these assessments. For the two hypothetical drugs, the calculated ICER for Japan was twice that of the UK, or 37% higher than that of the US. The implications of these findings for health technology assessment (HTA) are quite significant. Japan is now introducing an HTA system that is modeled on the UK system, using cost-utility analysis as a basis [31]. If the equivalent threshold is to be applied, the same drugs would then sell at lower prices in Japan than in the UK, due to differences in utility values that in turn differ due to different time preference rates.

While according to Hofstede time preference is culturally determined, there might be other influencing factors involved. In the economic literature, time preference is linked to a state’s level of economic development [32]. Time series data analysis for instance can show that for Taiwan and Japan, the time preference rate decreased up to a certain point during economic catch-up, with a further decline afterwards as the populations became more hedonistic [33]. Furthermore, individual factors might play a role as there is a potentially large variation in preferences within the same country. Low educational status for instance was identified as a key correlate of a high time preference which in turn contributes to unhealthy behavior such as smoking [34, 35]. Conversely, individuals with a long-term horizon are more likely to participate in higher education and adopt a healthier lifestyle [36]. The relationship between long-term orientation, cognitive function and health together with other outcomes has been documented in several studies [37, 38]. Golsteyn et al. for instance asked 13,606 Swedish children aged 13 whether they would prefer to receive $140 now or $1400 in 5 years. The study traced the children’s long-term achievements regarding education, fertility decisions, health indicators, labor market success, and income. The authors observed that impatient children perform worse in school and consequently earn less income, are more often unemployed, and more often depend on welfare benefits. Moreover, the study even reported that impatient children are more likely to die young, become obese, or become pregnant while still in their teens [39]. Subsequent research further demonstrated that impatient people are more likely to become involved in crimes later in life [40, 41].

While it is difficult to disentangle cultural and individual drivers of time preference, we believe that cultural factors shape preferences and should be accounted for in health economics. One implication of this debate is that the HTA concept of cost-utility analysis depends on cultural beliefs and values and cannot be easily transferred across cultures without significant adaptations. While we have looked at only two dimensions of Hofstede’s concept of culture, we acknowledge other cultural values. Hofstede himself defined four other pillars, namely power distance, individualism, masculinity, and indulgence. Religion is another important part of cultural values that either influence Hofstede’s dimensions or even constitute an independent pillar of culture impacting on choices of patients [42]. Future research could apply a more holistic approach to culture and relate it to health economics concepts.

Another limitation is that some of the EQ-5D-5L value sets were mapped from the EQ-5D-3L and those values are therefore not directly obtained preferences. We cannot rule out the possibility that the use of crosswalk value sets influences the results.

## Conclusion

We maintain that cultural beliefs determine utility values that are beneficial in cost-utility analysis and health technology assessments. Countries such as Japan are characterized by a long-term time horizon, or a low time preference rate. Cultural practices and beliefs are reflected in a concave shape of a country’s utility function. Such a curvature profile implies that incremental health gains are valued less. Accordingly, health-care interventions create different value in different cultural settings that should be accounted for in HTA. Consequently, our study further supports the accumulating literature that argues against a one-size-fits-all cost-effectiveness approach [43]. Instead, we maintain that a context-sensitive, multiple-criteria decision-making approach is warranted that should include values, cultural and country specific goals and goes beyond pure cost-effectiveness. Or, as it was expressed by Chalkidou et al. ‘[take] into account local values is the holy grail for country empowerment’ [44].
